# Self-Sustained Chaotic Jumping of Liquid Crystal Elastomer Balloon under Steady Illumination

**DOI:** 10.3390/polym15244651

**Published:** 2023-12-08

**Authors:** Xin Sun, Yuntong Dai, Kai Li, Peibao Xu

**Affiliations:** Department of Civil Engineering, Anhui Jianzhu University, Hefei 230601, China; xinsun1120@163.com (X.S.); daiytmechanics@ahjzu.edu.cn (Y.D.); kli@ahjzu.edu.cn (K.L.)

**Keywords:** liquid crystal elastomer, balloon, self-sustained chaotic jumping, bifurcation diagram, steady illumination

## Abstract

Self-sustained chaotic jumping systems composed of active materials are characterized by their ability to maintain motion through drawing energy from the steady external environment, holding significant promise in actuators, medical devices, biomimetic robots, and other fields. In this paper, an innovative light-powered self-sustained chaotic jumping system is proposed, which comprises a liquid crystal elastomer (LCE) balloon and an elastic substrate. The corresponding theoretical model is developed by combining the dynamic constitutive model of an LCE with Hertz contact theory. Under steady illumination, the stationary LCE balloon experiences contraction and expansion, and through the work of contact expansion between LCE balloon and elastic substrate, it ultimately jumps up from the elastic substrate, achieving self-sustained jumping. Numerical calculations reveal that the LCE balloon exhibits periodic jumping and chaotic jumping under steady illumination. Moreover, we reveal the mechanism underlying self-sustained periodic jumping of the balloon in which the damping dissipation is compensated through balloon contact with the elastic substrate, as well as the mechanism involved behind self-sustained chaotic jumping. Furthermore, we provide insights into the effects of system parameters on the self-sustained jumping behaviors. The emphasis in this study is on the self-sustained chaotic jumping system, and the variation of the balloon jumping modes with parameters is illustrated through bifurcation diagrams. This work deepens the understanding of chaotic motion, contributes to the research of motion behavior control of smart materials, and provides ideas for the bionic design of chaotic vibrators and chaotic jumping robots.

## 1. Introduction

Active materials have received extensive attention on account of their specific responsive properties. These materials include hydrogels, ionic gels, dielectric elastomers, thermosensitive polymer materials, and notably liquid crystal elastomers (LCEs) [1,2,3,4,5,6,7,8]. Furthermore, these materials have led to the engineering of diverse modes of self-sustained motion modes, each characterized by their own unique dynamics. These motion modes span from vibration [9,10,11], bending [12,13,14,15], rolling [16,17], torsion [18,19], stretching and contraction [20,21], to even swimming [22], oscillating [23,24,25], buckling [26,27,28,29,30], jumping [31,32,33], rotating [34], valving, or reversing [35,36]. Intricate nonlinear feedback mechanisms, including phenomena such as self-shading [26], the coupling of large deformations with chemical reactions [1,2], and the generation of photothermal surface tension gradients [37,38], make these self-sustained motion modes possible.

In recent years, LCEs, as a notable category of smart materials, have garnered substantial attention owing to their distinctive structural characteristics. Constituted by interconnected liquid crystal monomers, LCEs are remarkably responsive to external stimuli, such as light [6], heat [39], electricity [40], and magnetism [41]. When encountering these stimuli, LCEs undergo phase transitions or alterations in their molecular structures, bringing about significant macroscopic deformations [42]. Remarkably, upon withdrawal of the external stimuli, LCEs can revert to their original state. Thus, LCEs can be applied in many fields, such as soft robots [43], energy harvesting devices [44,45], wireless micro-nano machines [35], motors [46], active machines [47,48,49], etc.

Self-sustained motion systems actively harness external energy sources to maintain their non-equilibrium and sustained motions [50,51,52,53,54,55]. They typically behave with considerable robustness in terms of frequency and amplitude depending on the characteristics of the system parameters [6,7,8,56,57]. However, only very few scholars are currently conducting research on the phenomenon of self-sustained chaos; for instance, Xu et al. [33] proposed a self-sustained chaotic floating system that utilizes LCE balloons equipped with self-shading coatings under steady illumination. Similarly, Kumar et al. [58] engineered a self-sustained chaotic actuator based on LCE films doped with azobenzene and showcased the optically responsive behavior that drives the film motion under continuous illumination. Further research on self-sustained chaotic motion is essential as it contributes to the development of various fields, such as heart beating simulation and bionics [59], heart and brain chaos analysis [60,61], cardiovascular disease prevention [62], encrypted images [63], autonomous separation and stirrer [64,65], etc.

In order to open up a large number of application areas and functionalities, it is a necessity to build more self-sustained chaotic systems relying on active materials, so as to reveal their complex chaotic mechanisms and behaviors. This paper is dedicated to exploring and analyzing a distinct class of self-sustained chaotic jumping systems constituting an LCE balloon and an elastic substrate. Under steady illumination, the LCE balloon in our study underwent a transition from a static state to a self-sustained jumping state by actively drawing light energy from the surrounding environment. Different from the previous self-sustained floating system composed by a nonlinear spring and an LCE balloon [33], in which the damping dissipation of the system is compensated by the work of buoyancy varying with the radius of the balloon, this unique self-sustained mechanism proposed in this work leverages the contact expansion work of the balloon against an elastic substrate to counteract the damping dissipation, thereby sustaining its motion. The proposed self-jumping system is more adaptable and can move in different terrains and environments, including jumping on uneven ground. The movement of the system is more efficient, allowing it to move quickly and be able to cover longer distances in a shorter time. In addition, the system offers the capability to switch between periodic jumping and chaotic jumping via systematic adjustments of key parameters. This research facilitates a more profound comprehension of chaotic phenomena and broadens the application scope of chaos theory.

The remainder of this paper is structured as follows. Section 2 derives the dynamic governing equation based on the balloon jumping model and presents the solution method. In Section 3, two motion modes of the LCE balloon together with their mechanisms are introduced, and their mechanisms are explained. In Section 4, the effects of different system parameters on the motion modes are examined. Finally, Section 5 summarizes this paper.

## 2. Model and Theoretical Formulation

**Figure 1:**
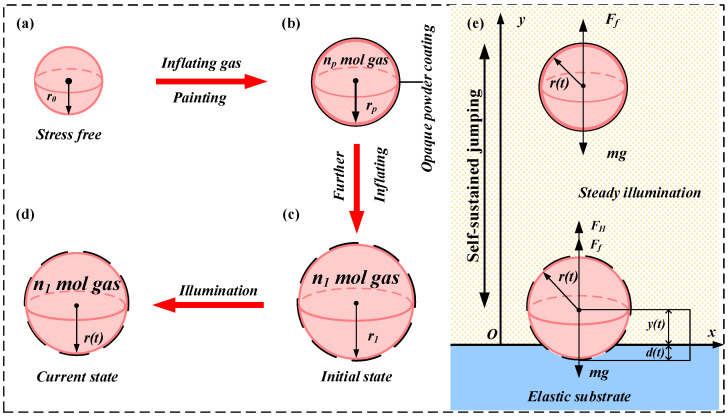
Schematics of the self-jumping LCE balloon under steady illumination. (**a**) Reference state of the stress-free LCE balloon with radius $r_{0}$. (**b**) The stress-free balloon is first inflated to the state with the painting radius $r_{p}$ and then painted by opaque powder coating. (**c**) Initial state of the LCE balloon attained after further inflation with initial radius $r_{1}$. (**d**) Current state of the LCE balloon under steady illumination with variable radius r(t). (**e**) Schematic of a self-jumping balloon with the variable radius r(t) under illumination.

In this section, a self-sustained chaotic jumping system is proposed, as illustrated in Figure 1, which comprises an LCE balloon and an elastic substrate and operates under illumination due to the self-shading effect. Based on the Hertz contact theory, the dynamic constitutive model of LCE, and the ideal gas equation, the governing equation for the self-sustained jumping of the LCE balloon and the deformation theory of the LCE balloon are acquired.

### 2.1. Governing Equation of Self-Jumping System

Figure 1 plots the nonlinear dynamics model of the LCE balloon, where the variable radius of the balloon is r(t) and the mass m of the balloon remains constant. In response to the steady illumination, a stationary LCE balloon positioned on an elastic substrate experiences a repetitive cycle of contraction and expansion, eventually leading to the jump from the elastic substrate. The damping effect is counteracted by the work generated when the balloon comes into contact with the elastic substrate and expands. This interaction results in a self-sustained jumping motion of the balloon. The pre-processing steps for the LCE balloon are described in Figure 1a–d. In Figure 1a, we consider the stress-free state of the balloon as the reference state. In this state, we use $r_{0}$ to represent the radius, $\rho_{L}$ to denote the mass density of the LCE balloon and $h_{0}$ to represent the thickness of the stress-free balloon. As shown in Figure 1b, a certain amount of gas $n_{p}$ is inflated into the LCE balloon to enlarge its radius up to the painting radius $r_{p}$, and then an opaque powder coating is applied to its surface. In Figure 1c, the balloon reaches equilibrium after further inflation, which is treated as the initial state, where the amount of gaseous substance for the gas inside the balloon is $n_{1}$ and the balloon radius is $r_{1}$. Figure 1d depicts the current state of the balloon during its motion under steady illumination, where the balloon radius is r(t). Assuming that the LCE material is incompressible, the volume of the LCE balloon stays constant at $V_{L}$. Considering that the balloon thickness is much smaller than the radius, the thickness of the LCE balloon can be calculated as $h=\frac{V_{L}}{4\pi r^{2}}$. Since the LCE balloon contracts in the presence of illumination and expands in the absence of illumination, when the variable radius $r\left(t\right)>r_{p}$, the opaque powder coating is held open and the balloon contracts; when the variable radius $\;r\left(t\right)\leq r_{p}$, the opaque powder coating remains closed and the balloon expands. When the balloon is in contact with the elastic substrate and expands, the negative work dissipated by the system damping is compensated, and the self-sustained jumping can be achieved via adjusting the system parameters.

As shown in Figure 1e, two kinds of force analysis are present for the balloon due to the influence of Hertz contact force. When the balloon is in contact with the elastic substrate, it is mainly subjected to Hertz contact force $F_{H}$, gravity mg, and air resistance $F_{f}=-\beta \dot{y}$, where β is the air damping coefficient and $\dot{y}$ denotes the velocity of the LCE balloon. When the balloon is not in contact with the elastic substrate, it is only subjected to gravity mg and air resistance $F_{f}$. Therefore, the Hertz contact force $F_{H}$ can be described as follows [66]:

(1) FH=0y>rtFH=43Er12d32y≤rt

where E is the equivalent elastic modulus, and d refers to the indentation depth expressed as d=r(t)−y(t) for $y\leq r\left(t\right)$.

The dynamic governing equation of the LCE balloon can be stated as follows:

(2) $$m\ddot{y}=-mg+F_{f}+F_{H}$$

where $\ddot{y}$ denotes the acceleration of the LCE balloon. Generally, the force of air resistance depends on the aerodynamic drag coefficient and frontal area, which are characteristic of the object, the density of the medium, and the square of the velocity [67]. For the sake of simplification, the jumping of the LCE balloon studied in this paper is assumed to be at a low speed, and the air resistance is assumed as $F_{f}$ = $-\beta \dot{y}$, where β and $\dot{y}$ are the air damping coefficient and velocity of the LCE balloon, respectively.

Therefore, Equation (2) can be rewritten as

(3) y¨=−g−βy˙my>rty¨=−g−βy˙m+43Er12(r−y)32my≤rt

### 2.2. Dynamic of the Spherical LCE Balloon

**Figure 2:**
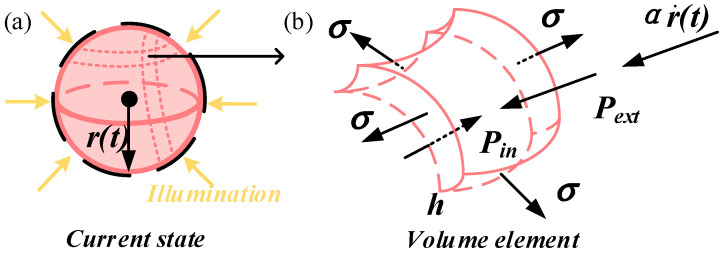
Schematics of force analysis of the LCE balloon. (**a**) Schematic diagram of LCE balloon deformation under the current state. (**b**) Force analysis of a small volume element.

To simplify the model, we ignore the effect of gravitational acceleration. Figure 2a draws a schematic diagram of LCE balloon deformation under the current state. We take a volume element as shown in Figure 2b for force analysis. It is evident that the volume element is mainly subjected to the internal gas pressure $P_{in}$, the external pressure $P_{ext}$, the damping force, and the Laplace pressure $P_{L}$ from the surface tension. According to the Newtonian dynamics, the governing equation for the dynamics of the LCE balloon can be formulated as

(4) $$P_{in}-P_{ext}-P_{L}-\alpha \frac{dr}{dt}=\rho_{L}h\frac{d^{2}r}{dt^{2}}$$

where $\frac{d^{2}r}{dt^{2}}$ and $\frac{dr}{dt}$ correspond to the radial acceleration and velocity, respectively. Additionally, ρ and h are used to represent the mass density and thickness of the LCE balloon, while α signifies the damping coefficient associated with the radial deformation of the LCE balloon.

On the assumption that the gas inside the balloon follows the ideal gas model with equation of state $P_{in}V=n_{1}RT$ and V can be described as $V=\frac{4}{3}\pi r^{3}$, we can consequently express the internal pressure of the balloon as follows:

(5) $$P_{in}=\frac{3n_{1}RT}{4\pi r^{3}}$$

where $n_{1}$ stands for the amount of gaseous substance, *R* denotes the ideal gas constant, and *T* represents the thermodynamic temperature of the ideal gas.

The Laplace pressure arising from membrane tension can be stated as

(6) $$P_{L}=\frac{2\sigma h}{r}$$

where the principal stress σ on the balloon surface can be represented as $\sigma =E_{eff}\varepsilon$.

Therefore, the Laplace pressure can be rewritten as

(7) $$P_{L}=\frac{2E_{eff}\varepsilon h}{r}$$

Substituting Equations (5) and (7) into Equation (4) yields

(8) $$\frac{3n_{1}RT}{4\pi r^{3}}-P_{ext}-\frac{2E_{eff}\varepsilon h}{r}-\alpha \frac{dr}{dt}=\rho_{L}h\frac{d^{2}r}{dt^{2}}$$

in which

(9) $$\varepsilon =\frac{r-r_{0}(1+\varepsilon_{L})}{r_{0}(1+\varepsilon_{L})}$$

with $\varepsilon_{L}$ being the effective light-driven strain of the LCE balloon. $\varepsilon_{L}=-C_{0}\phi (t)$ is present, where ϕ(t) is the number fraction of bent *cis* isomers induced by light-driven *trans*-to-*cis* excitation and $C_{0}$ is the contraction coefficient, which is positive when the LCE balloon contracts. Generally, the *trans*-to-*cis* isomerization of LCE can be induced by UV or laser with a wavelength less than 400 nm [68]. Therefore, the *cis* number fraction depends on the thermal excitation from trans to *cis*, thermal relaxation from *cis* to trans, and photo-driven trans-to-cis isomerization. Compared with the photo-driven *trans*-to-*cis* isomerization, the thermal excitation from *cis* to trans is usually negligible [68].

To proceed with our discussions, we focus on determining the *cis* number fraction ϕ(t) within the LCE balloon. It can be characterized as follows [68,69]:

(10) $$\frac{\partial \phi }{\partial t}=\eta_{0}I(1-\phi )-\frac{\phi }{T_{0}}$$

where $T_{0}$ is the thermal relaxation time from *cis* to *trans*, I refers to the light intensity, and $\eta_{0}$ denotes the light absorption coefficient. By solving Equation (10), the *cis* number fraction ϕ(t) is obtained:

(11) $$\phi (t)=\frac{\eta_{0}T_{0}I}{\eta_{0}T_{0}I+1}+\left(\phi_{0}-\frac{\eta_{0}T_{0}I}{\eta_{0}T_{0}I+1}\right)\exp\left[-\frac{t}{T_{0}}(\eta_{0}T_{0}I+1)\right]$$

where $\phi_{0}$ represents the initial *cis* number fraction.

There exist three distinct cases for the *cis* number fraction, and we enumerate each of them sequentially. For case I, which pertains to the LCE balloon under illumination with an initial *cis* number fraction $\phi_{0}=0$, Equation (11) can be simplified as

(12) $$\phi (t)=\frac{\eta_{0}T_{0}I}{\eta_{0}T_{0}I+1}\left\{1-\exp\left[-\frac{t_{1}}{T_{0}}\left(\eta_{0}T_{0}I+1\right)\right]\right\}$$

For case II, where the LCE balloon in the illumination state is switched from the dark state with a transient *cis* number fraction of $\phi_{0}=\phi_{dark}$, Equation (11) can be simplified as

(13) $$\phi (t)=\frac{\eta_{0}T_{0}I}{\eta_{0}T_{0}I+1}+\left(\phi_{dark}-\frac{\eta_{0}T_{0}I}{\eta_{0}T_{0}I+1}\right)\exp\left[-\frac{t_{2}}{T_{0}}\left(\eta_{0}T_{0}I+1\right)\right]$$

For case III in which the LCE balloon in the dark state (I=0) is switched from the illumination state with a transient *cis* number fraction of $\phi_{0}=\phi_{illum}$, Equation (11) can be simplified as

(14) $$\phi (t)=\phi_{illum}\exp(-\frac{t_{3}}{T_{0}})$$

Following Equations (12)–(14), the *cis* number fraction can be easily estimated, according to which the effective light-driven strain $\varepsilon_{L}$ can be obtained. Then, when the external pressure $P_{ext}$ is given, the balloon radius r(t) can be derived by Equation (8).

### 2.3. Nondimensionalization

To provide a general description of the jumping phenomenon while eliminating dimensional effects, we opt for a dimensionless approach to the system parameters. For the governing equation of jumping system, the corresponding dimensionless parameters are as follows: $\bar{y}=\frac{y}{r_{0}}$, $\bar{\dot{y}}=\frac{\dot{y}T_{0}}{r_{0}}$, $\bar{\ddot{y}}=\frac{\ddot{y}T_{0}{}^{2}}{r_{0}}$, $\bar{r}=\frac{r}{r_{0}}$, $\bar{E}=\frac{ET_{0}{}^{2}r_{0}}{m}$, $\bar{\beta }=\frac{\beta T_{0}}{m}$, $\bar{t}=\frac{t}{T_{0}}$, and $\bar{g}=\frac{gT_{0}{}^{2}}{r_{0}}$.

Equation (3) can be expressed dimensionlessly as

(15) y¨¯=−g¯−β¯y˙¯y¯>r¯t¯y¨¯=−g¯−β¯y˙¯+43E¯r¯12(r¯−y¯)32y¯≤rt¯

For the governing equation of the balloon vibration, the corresponding dimensionless parameters are as follows: ${\bar{P}}_{L}=\frac{P_{L}}{E_{eff}}$, ${\bar{P}}_{ext}=\frac{p_{ext}}{E_{eff}}$, ${\bar{n}}_{1}=\frac{3n_{1}RT}{4\pi E_{eff}r_{0}{}^{3}}$, ${\bar{V}}_{L}=\frac{V_{L}}{4\pi r_{0}{}^{3}}$, $\bar{\alpha }=\frac{\alpha r_{0}}{E_{eff}T_{0}}$, ${\bar{\rho }}_{L}=\frac{\rho r_{0}{}^{2}}{E_{eff}T_{0}{}^{2}}$, and $\bar{I}=\eta_{0}T_{0}I$.

Equation (8) can be nondimensionalized as

(16) $$\frac{d^{2}\bar{r}}{d{\bar{t}}^{2}}=\frac{{\bar{n}}_{1}}{{\bar{\rho }}_{L}{\bar{V}}_{L}\bar{r}}-\frac{{\bar{p}}_{ext}{\bar{r}}^{2}}{{\bar{\rho }}_{L}{\bar{V}}_{L}}-2\frac{\bar{r}-1-\varepsilon_{L}}{\bar{r}{\bar{\rho }}_{L}\left(1+\varepsilon_{L}\right)}-\frac{\bar{\alpha }{\bar{r}}^{2}}{{\bar{\rho }}_{L}{\bar{V}}_{L}}\frac{d\bar{r}}{d\bar{t}}$$

For case I, applying the dimensionless approach to Equation (12) results in

(17) $$\phi (\bar{t})=\frac{\bar{I}}{\bar{I}+1}\left\{1-\exp\left[-{\bar{t}}_{1}\left(\bar{I}+1\right)\right]\right\}$$

In the context of case II, Equation (13) can be made dimensionless as follows:

(18) $$\phi (\bar{t})=\frac{\bar{I}}{\bar{I}+1}+\left(\phi_{dark}-\frac{\bar{I}}{\bar{I}+1}\right)\exp\left[-{\bar{t}}_{2}\left(\bar{I}+1\right)\right]$$

For case III, the dimensionless representation of Equation (14) is

(19) $$\phi (\bar{t})=\phi_{illum}\exp(-{\bar{t}}_{3})$$

Up to this point, we can obtain the radius of the balloon by using Equation (16). Next, by substituting the radius of the balloon into Equation (15), we calculate the displacement of the balloon. Since Equation (15) lacks an analytical solution, we need to employ the fourth-order Runge–Kutta method for solving it. The Runge–Kutta method is as follows:

(20) $$y\left(\bar{t}+h\right)=y(\bar{t})+\frac{1}{6}(L_{1}+2L_{2}+2L_{3}+L_{4})$$

where

(21) $$\left\{\begin{array}{l} L_{1}=hf(\bar{t},y)\\ L_{2}=hf(\bar{t}+\frac{1}{2}h,y+\frac{1}{2}L_{1})\\ L_{3}=hf(\bar{t}+\frac{1}{2}h,y+\frac{1}{2}L_{2})\\ L_{4}=hf(\bar{t}+h,y+L_{3}) \end{array}\right.$$

and h represents the time interval; we adopt a time step of h=0.0001 in the computation.

## 3. Two Jumping Modes and Mechanisms

In accordance with the above theoretical model, this section proposes two motion modes through numerical calculation and explains the mechanisms underlying the two motion modes.

### 3.1. Two Jumping Modes

**Figure 3:**
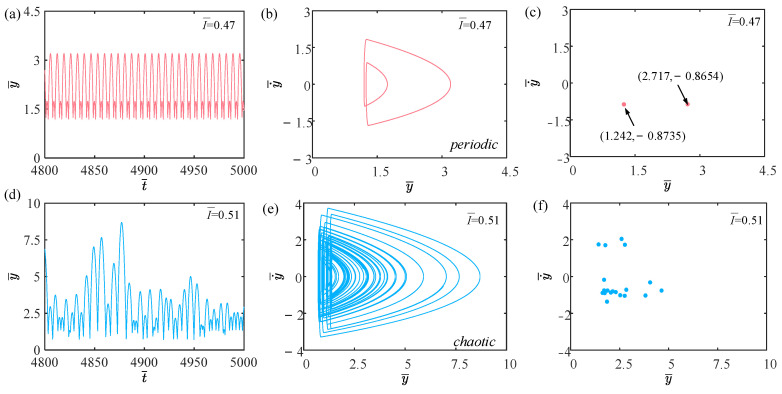
Two typical motion modes: periodic jumping and chaotic jumping. (**a**) Time history curve with $\bar{I}=0.47$. (**b**) Phase diagram with $\bar{I}=0.47$. (**c**) Poincare map with $\bar{I}=0.47$. (**d**) Time history curve with $\bar{I}=0.51$. (**e**) Phase diagram with $\bar{I}=0.51$. (**f**) Poincare map with $\bar{I}=0.51$.

Figure 3 shows the time history curves, phase diagrams, and Poincare maps with different light intensities. Video S1 demonstrates periodic jumping of the LCE balloon. Video S2 demonstrates chaotic jumping of the LCE balloon. The parameters, except for light intensity, are set to $C_{0}=0.4$, ${\bar{V}}_{L}=0.8$, ${\bar{P}}_{ext}=0.3$, $\bar{\beta }=0.05$, ${\bar{n}}_{1}=0.6$, ${\bar{\rho }}_{L}=1$, $\bar{\alpha }=0.1$, $\bar{E}=10,000$, $\bar{g}=0.8$, and ${\bar{R}}_{p}=1.2$. The time history curve in Figure 3a contains two different peaks, indicating that the balloon jumps alternatively at two distinct amplitudes. As displayed in Figure 3b, there appear only two regular trajectories on the phase diagram, suggesting that the balloon motion is regular. There are also only two discrete points on the Poincare map in Figure 3c. The above calculation outcomes show that the balloon is in a periodic jumping at this time. As presented in Figure 3d, the balloon lacks a stable amplitude on the time history curve, the trajectories on the phase diagram are complex and disordered in Figure 3e, and the points on the Poincare map have no regularity in Figure 3f. Such calculation results imply that the balloon is in a chaotic jumping. To elucidate the origin of both sustained periodic jumping and chaotic jumping, we will delve into the mechanisms underlying these two self-sustained jumping modes in the following section.

### 3.2. Mechanisms of the Two Jumping Modes

**Figure 4:**
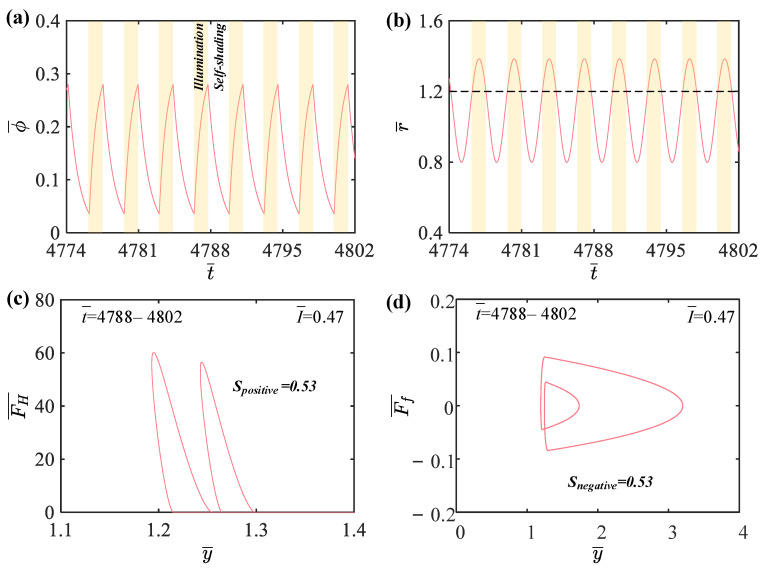
Mechanism of the periodic self-sustained jumping of the LCE balloon. (**a**) Time variation of cis number fraction in time span $\bar{t}=4774–4802$. (**b**) Time variation of balloon radius in time span $\bar{t}=4774–4802$. (**c**) Dependence of Hertz contact force on balloon displacement with $\bar{I}=0.47$ in time span $\bar{t}=4788–4802$. (**d**) Dependence of air resistance on balloon displacement with $\bar{I}=0.47$ in time span $\bar{t}=4788–4802$.

In this section, we further explore the mechanisms of self-sustained periodic jumping and chaotic jumping of the LCE balloon. Figure 4 provides several relevant data plots explaining the mechanism of periodic jumping for the LCE balloon, with the following system parameter values: $C_{0}=0.4$, ${\bar{V}}_{L}=0.8$, ${\bar{P}}_{ext}=0.3$, $\bar{\beta }=0.05$, ${\bar{n}}_{1}=0.6$, ${\bar{\rho }}_{L}=1$, $\bar{\alpha }=0.1$, $\bar{E}=10,000$, $\bar{g}=0.8$, $\bar{I}=0.47$, and ${\bar{R}}_{p}=1.2$. As shown in Figure 4a, the cis number fraction of the balloon varies over time under steady illumination. The balloon radius also varies as the cis number fraction varies. As the powder coating on the balloon surface is turned on or off, the cis number fraction varies periodically with time, which induces a periodic increase or decrease in the balloon radius, as depicted in Figure 4b. As a consequence, when the balloon is in contact with the elastic substrate and expands, the Hertz contact force can compensate for the damping dissipation of the system, thus maintaining a stable self-sustained jumping. From the previous section, when the light intensity is 0.47, the balloon sustains a periodic jumping, which means that the balloon contacts the elastic substrate twice in one period. As illustrated in Figure 4c, when $\bar{t}=4788-4802$, the balloon contacts the elastic substrate twice, and the positive work done by the Hertz contact force is $S_{positive}=0.53$. In Figure 4d, the air resistance does negative work of $S_{negative}=0.53$ over the same time span. Therefore, the balloon will jump periodically when the net work of the Hertz contact force is identical to the negative work of the damping dissipation over the same time span.

**Figure 5:**
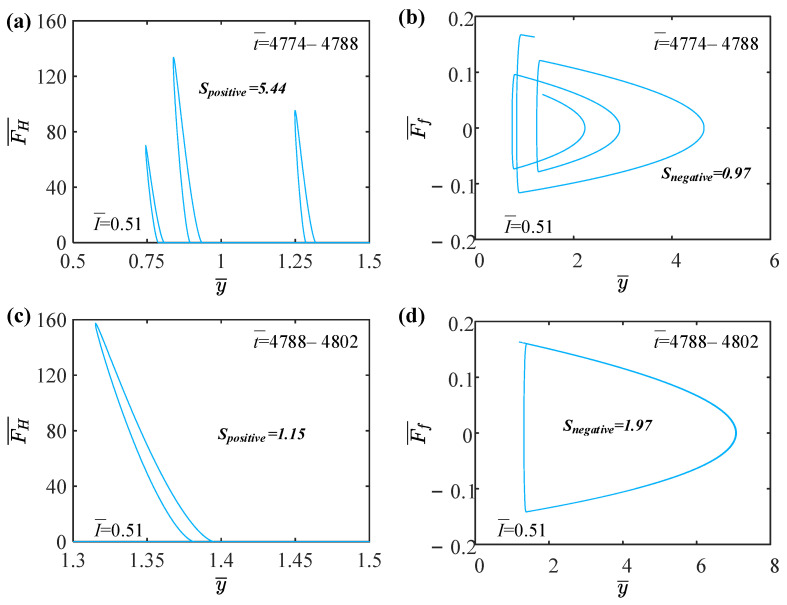
Mechanism of the self-sustained chaotic jumping of the LCE balloon. (**a**) Dependence of Hertz contact force on balloon displacement with $\bar{I}=0.51$ in time span $\bar{t}=4774-4788$. (**b**) Dependence of air resistance on balloon displacement with $\bar{I}=0.51$ in time span $\bar{t}=4774-4788$. (**c**) Dependence of Hertz contact force on balloon displacement with $\bar{I}=0.51$ in time span $\bar{t}=4788-4802$. (**d**) Dependence of air resistance on balloon displacement with $\bar{I}=0.51$ in time span $\bar{t}=4788-4802$.

Figure 5 illustrates several informative data plots that elucidate the process of chaotic jumping in the LCE balloon, with the corresponding system parameter values being $C_{0}=0.4$, ${\bar{V}}_{L}=0.8$, ${\bar{P}}_{ext}=0.3$, $\bar{\beta }=0.05$, ${\bar{n}}_{1}=0.6$, ${\bar{\rho }}_{L}=1$, $\bar{\alpha }=0.1$, $\bar{E}=10,000$, $\bar{g}=0.8$, $\bar{I}=0.51$, and ${\bar{R}}_{p}=1.2$. It is apparent from Figure 5a that when $\bar{t}=4774-4788$, the balloon collides with the elastic substrate several times, and the positive work done by the Hertz contact force is $S_{positive}=5.44$. As displayed in Figure 5b, the trajectory in the figure is not continuous due to the selection of the time span, but it does not affect the analysis. The air resistance performs negative work of $S_{negative}=0.53$ over the same time span, namely, $S_{positive}>S_{negative}$. As with the above analysis for $\bar{t}=4774-4788$, when $\bar{t}=4788-4802$, the positive work of the Hertz contact force is 1.15, and the negative work of the air resistance is 1.97, that is, $S_{positive}$ is less than $S_{negative}$. Accordingly, chaos occurs when the net work of the Hertz contact force is not equivalent to the negative work of the damping force within the same time span.

## 4. Effects of System Parameters on Sustained Jumping

In this section, the control variable method is adopted to investigate the effects of different system parameters on the jumping modes of the LCE balloon, which are systematically discussed by plotting time history curves, phase diagrams, Poincare maps, and bifurcation diagrams.

### 4.1. Effect of Light Intensity

**Figure 6:**
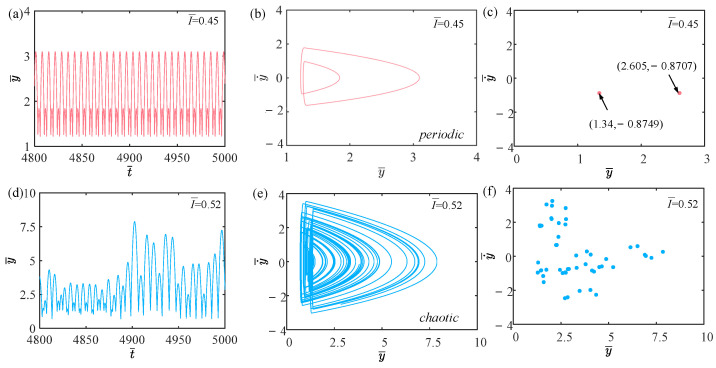
Effects of different dimensionless light intensities on the self-sustained jumping of the balloon. (**a**) Time history curve at $\bar{I}=0.45$. (**b**) Phase diagram at $\bar{I}=0.45$. (**c**) Poincare map at $\bar{I}=0.45$. (**d**) Time history curve at $\bar{I}=0.52$. (**e**) Phase diagram with $\bar{I}=0.52$. (**f**) Poincare map at $\bar{I}=0.52$.

**Figure 7:**
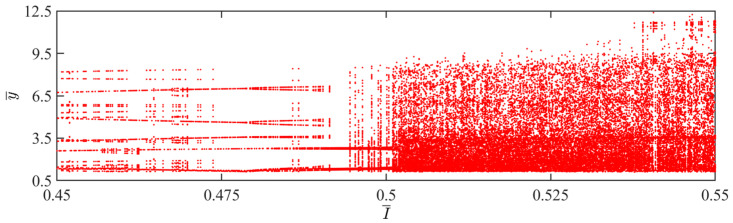
Bifurcation diagram with varying dimensionless light intensities.

This section mainly discusses the effect of light intensity on the self-sustained jumping of the LCE balloon. Apart from light intensity, the other system parameters are assigned the following values: $C_{0}=0.4$, ${\bar{V}}_{L}=0.8$, ${\bar{P}}_{ext}=0.3$, $\bar{\beta }=0.05$, ${\bar{n}}_{1}=0.6$, ${\bar{\rho }}_{L}=1$, $\bar{\alpha }=0.1$, $\bar{E}=10,000$, $\bar{g}=0.8$, and ${\bar{R}}_{p}=1.2$. Figure 6 provides the time history curves, phase diagrams, and Poincare maps for two different dimensionless light intensities, while Figure 7 presents the bifurcation diagram corresponding to light intensity varying from 0.45 to 0.55. Video S3 shows the effect of light intensity on the jumping mode of the LCE balloon. When the light intensity is 0.45, two peaks appear on the time history curve in Figure 6a, indicating that the balloon alternates between two stable amplitudes. The trajectory in the corresponding phase diagram depicted in Figure 6b exhibits repetitive motion along a specific course. And the corresponding Poincare map in Figure 6c is displayed as two discrete points, which are located at specific locations in phase space. The above phenomena suggest that the balloon is in a periodic jumping at this time. It can be observed from Figure 6d that the balloon has no stable amplitude when the light intensity is 0.52, i.e., the balloon is unable to maintain a steady jumping. Many complex trajectories appear on the corresponding phase diagram in Figure 6e, and these trajectories have no apparent periodicity. There appear many discrete points on the corresponding Poincare map in Figure 6f with no obvious periodicity. These phenomena reflect that the balloon is in a chaotic jumping at this time.

The aforementioned transition in the jumping modes is attributed to variations in light intensity, which directly affect the balloon radius, consequently altering the magnitude of the Hertz contact force. Chaos occurs when the work done by the Hertz contact force differs from the energy dissipated by damping. According to Figure 7, when the light intensity is between 0.45 and 0.5, the balloon maintains a periodic jumping. However, when the light intensity exceeds 0.5, the balloon predominantly exhibits a chaotic jumping.

### 4.2. Effect of Contraction Coefficient

**Figure 8:**
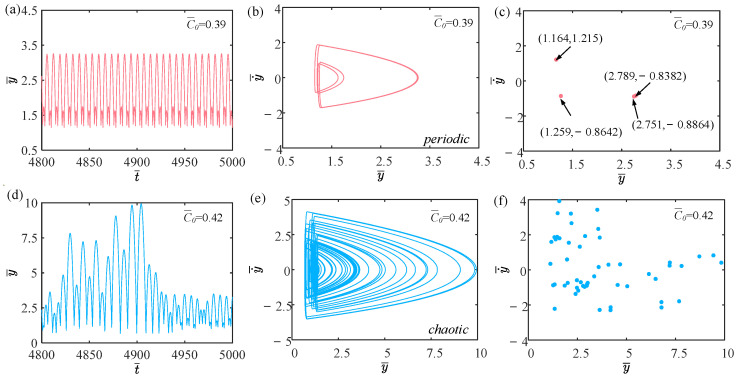
Effects of different dimensionless contraction coefficients on the self-sustained jumping of the balloon. (**a**) Time history curve at ${\bar{C}}_{0}=0.39$. (**b**) Phase diagram at ${\bar{C}}_{0}=0.39$. (**c**) Poincare map at ${\bar{C}}_{0}=0.39$. (**d**) Time history curve at ${\bar{C}}_{0}=0.42$. (**e**) Phase diagram with ${\bar{C}}_{0}=0.42$. (**f**) Poincare map at ${\bar{C}}_{0}=0.42$.

**Figure 9:**
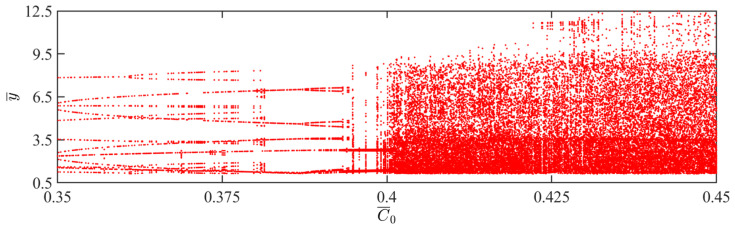
Bifurcation diagram with varying dimensionless contraction coefficients.

In this section, we delve into the effect of contraction coefficients on the self-sustained jumping of the LCE balloon, with other system parameters set as follows: $\bar{I}=0.5$, ${\bar{V}}_{L}=0.8$, ${\bar{P}}_{ext}=0.3$, $\bar{\beta }=0.05$, ${\bar{n}}_{1}=0.6$, ${\bar{\rho }}_{L}=1$, $\bar{\alpha }=0.1$, $\bar{E}=10,000$, $\bar{g}=0.8$, and ${\bar{R}}_{p}=1.2$. The time history curves, phase diagrams, and Poincare maps for two dimensionless contraction coefficients are found in Figure 8, accompanied by Figure 9, which is the corresponding bifurcation diagram as the contraction coefficient ranges from 0.35 to 0.45. Video S4 demonstrates the effect of the contraction coefficient on the jumping mode of the LCE balloon. The analysis of the calculation outcomes is similar to Section 4.1. As observed in Figure 8a–c, when the contraction coefficient is 0.39, the balloon alternates between two stable amplitudes, and the corresponding phase diagram and Poincare diagram indicate that the balloon is in a periodic motion mode. For Figure 8d–f, when the contraction coefficient is 0.42, the balloon is unable to maintain a steady jump, and the corresponding phase diagram and Poincare diagram suggest that the balloon is in a chaotic jumping mode.

With the increase in the contraction coefficient, the magnitude of the balloon deformation also increases. This alters the magnitude of the Hertz contact force. If the work performed by the Hertz contact force is not equal to the damping dissipation, chaos occurs. As shown in Figure 9, when the contraction coefficient increases to 0.4, the jumping mode of the balloon is predominantly chaotic.

### 4.3. Effect of Balloon Volume

**Figure 10:**
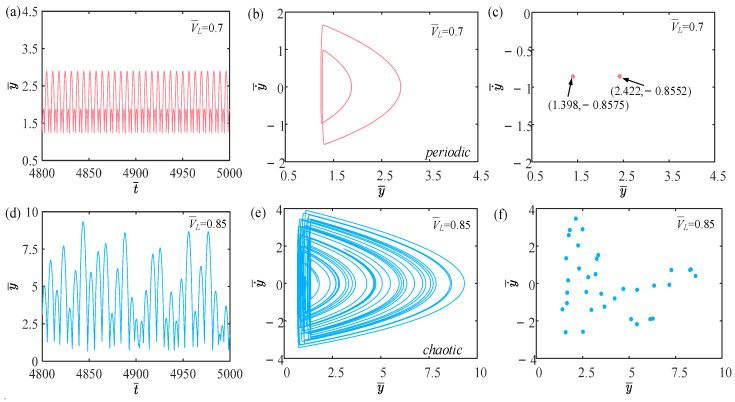
Effects of different dimensionless balloon volumes on the self-sustained jumping of the balloon. (**a**) Time history curve at ${\bar{V}}_{L}=0.7$. (**b**) Phase diagram at ${\bar{V}}_{L}=0.7$. (**c**) Poincare map at ${\bar{V}}_{L}=0.7$. (**d**) Time history curve at ${\bar{V}}_{L}=0.85$. (**e**) Phase diagram with ${\bar{V}}_{L}=0.85$. (**f**) Poincare map at ${\bar{V}}_{L}=0.85$.

**Figure 11:**
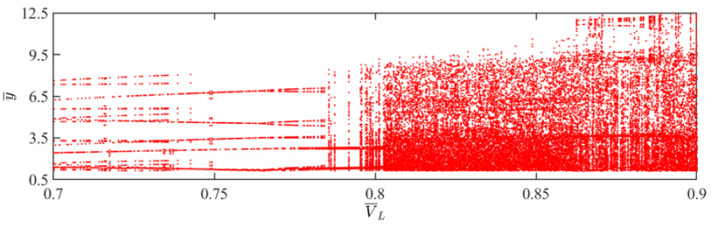
Bifurcation diagram with varying dimensionless balloon volumes.

In this section, we examine how the balloon volume influences the self-sustained jumping of the LCE balloon, in which the other system parameters are set as follows: $C_{0}=0.4$, $\bar{I}=0.5$, ${\bar{P}}_{ext}=0.3$, $\bar{\beta }=0.05$, ${\bar{n}}_{1}=0.6$, ${\bar{\rho }}_{L}=1$, $\bar{\alpha }=0.1$, $\bar{E}=10,000$, $\bar{g}=0.8$, and ${\bar{R}}_{p}=1.2$. Figure 10 provides the time history curves, phase diagrams, and Poincare maps for two distinct dimensionless balloon volumes, and the bifurcation diagram corresponding to balloon volume varying from 0.7 to 0.9 is depicted in Figure 11. Video S5 shows the effect of balloon volume on the jumping mode of the LCE balloon. The outcome analysis remains similar to Section 4.1. According to Figure 10a–c, when the balloon volume is 0.7, the balloon maintains a stable amplitude, with two regular trajectories on the corresponding phase diagram and only two points on the Poincare map. These are all indicative of the periodic jumping of the balloon. As observed in Figure 10d–f, the lack of a stable amplitude, the irregular trajectories on the corresponding phase diagram, and the several irregular points on the Poincare map indicate that the balloon is experiencing a chaotic jumping.

The change in the balloon radius increases with the increase in the balloon volume, thus changing the magnitude of the Hertz contact force. When the net work is not equal to the damping dissipation, chaos emerges. As illustrated in the bifurcation diagram of Figure 11, when the balloon volume grows to 0.8, the jumping mode of the balloon is mostly chaotic.

### 4.4. Effect of External Pressure

**Figure 12:**
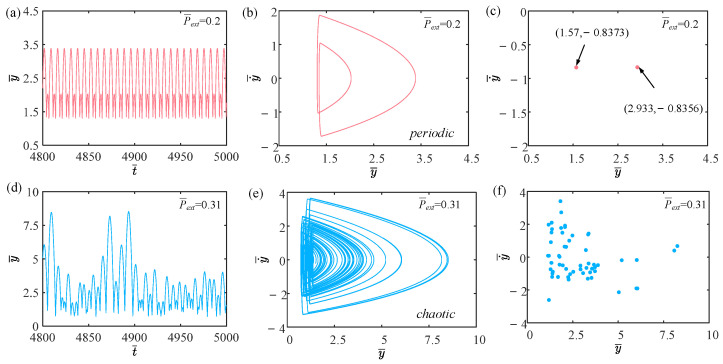
Effects of different dimensionless external pressures on the self-sustained jumping of the balloon. (**a**) Time history curve at ${\bar{P}}_{ext}=0.2$. (**b**) Phase diagram at ${\bar{P}}_{ext}=0.2$. (**c**) Poincare map at ${\bar{P}}_{ext}=0.2$. (**d**) Time history curve at ${\bar{P}}_{ext}=0.31$. (**e**) Phase diagram with ${\bar{P}}_{ext}=0.31$. (**f**) Poincare map at ${\bar{P}}_{ext}=0.31$.

**Figure 13:**
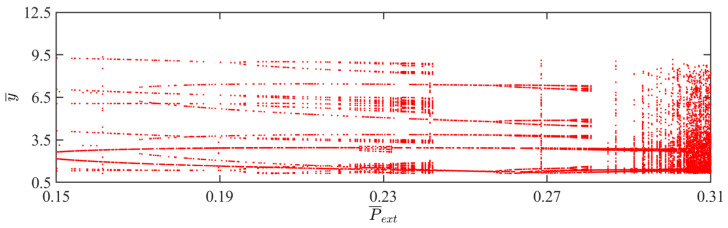
Bifurcation diagram with varying dimensionless external pressure.

This section mainly focuses on the effect of external pressure on the self-sustained jumping of the LCE balloon, and the other system parameters are set to $C_{0}=0.4$, $\bar{I}=0.5$, ${\bar{V}}_{L}=0.8$, $\bar{\beta }=0.05$, ${\bar{n}}_{1}=0.6$, ${\bar{\rho }}_{L}=1$, $\bar{\alpha }=0.1$, $\bar{E}=10,000$, $\bar{g}=0.8$, and ${\bar{R}}_{p}=1.2$. The time history curves, phase diagrams, and Poincare maps for two distinct dimensionless external pressures are presented in Figure 12, along with the bifurcation diagram corresponding to external pressure varying from 0.15 to 0.31 being depicted in Figure 13. Video S6 demonstrates the effect of external pressure on the jumping mode of the LCE balloon. The analysis is still similar to Section 4.1. It is clearly observed from Figure 12a–c that when the external pressure is 0.2, the balloon jumps alternately between two amplitudes, the trajectories on the phase diagram are regular, and there exist only two fixed points on the Poincare map. These outcomes imply that the jumping of the balloon is periodic. Furthermore, in Figure 12d–f, when the external pressure is 0.31, the jumping of the balloon has no stable amplitude, there appear no repeated trajectories on the corresponding phase diagram, and the points on the corresponding Poincare diagram are random, all reflecting that the balloon is in a chaotic jumping.

The alteration in the jumping modes of the balloon is attributed to variations in the deformation period of the balloon in response to fluctuations in the external pressure, which further affects the Hertz contact force. Chaos arises when the net work executed by the Hertz contact force is not in balance with the damping dissipation of the system. As displayed in Figure 13, the balloon exhibits periodic jumping when the light intensity is within the interval of 0.15 and 0.2, as well as the interval of 0.24 and 0.26. The jumping mode of the balloon switches between periodic and chaotic in the remaining intervals.

### 4.5. Effect of Damping Coefficient

**Figure 14:**
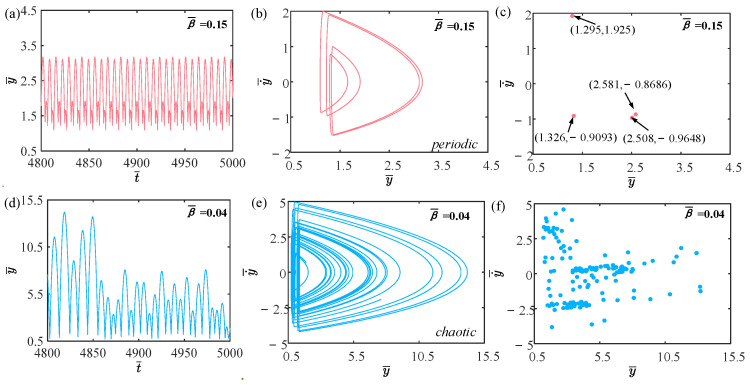
Effects of different dimensionless damping coefficients on the self-sustained jumping of the balloon. (**a**) Time history curve at ${\bar{\beta }}^{}=0.15$. (**b**) Phase diagram at ${\bar{\beta }}^{}=0.15$. (**c**) Poincare map at ${\bar{\beta }}^{}=0.15$. (**d**) Time history curve at ${\bar{\beta }}^{}=0.04$. (**e**) Phase diagram with ${\bar{\beta }}^{}=0.04$. (**f**) Poincare map at ${\bar{\beta }}^{}=0.04$.

**Figure 15:**
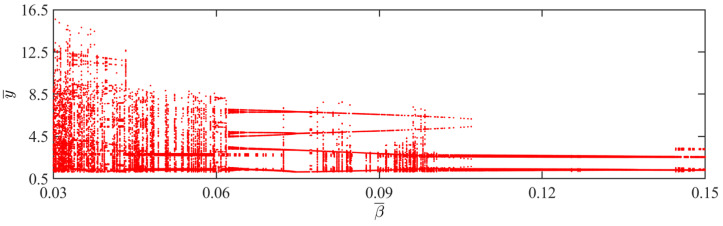
Bifurcation diagram with varying dimensionless damping coefficient.

The focus of this section is on the effect of the damping coefficient on the self-sustained jumping of the LCE balloon, in which the other system parameters are set to $C_{0}=0.4$, $\bar{I}=0.5$, ${\bar{V}}_{L}=0.8$, ${\bar{P}}_{ext}=0.3$, ${\bar{n}}_{1}=0.6$, ${\bar{\rho }}_{L}=1$, $\bar{\alpha }=0.1$, $\bar{E}=10,000$, $\bar{g}=0.8$, and ${\bar{R}}_{p}=1.2$. The time history curves, phase diagrams, and Poincare maps for two distinct dimensionless damping coefficients are depicted in Figure 14. Similarly, a bifurcation diagram of the dimensionless damping coefficient ranging from 0.03 to 0.15 is plotted in Figure 15. Video S7 demonstrates the effect of the damping coefficient on the jumping mode of the LCE balloon. The outcome analysis remains similar to Section 4.1. According to Figure 14a–c, when the damping coefficient is 0.15, the amplitude of the balloon is observed to remain stable, the trajectories on the phase diagram exhibit orderliness, and only four fixed points are present on the Poincare map. The above observations suggest that the jumping mode of the balloon is periodic for ${\bar{\beta }}^{}=0.15$. As illustrated in Figure 14d–f, when the damping coefficient is 0.04, the jumping of the balloon has no stable amplitude, and there are displayed many disordered trajectories on the corresponding phase diagram and many random points on the corresponding phase diagram. These outcomes imply that the jumping mode of the balloon is chaotic.

As the damping coefficient varies, the rate of energy dissipation in the system also varies. Chaos takes place when the energy input to the system and the energy dissipated by the system damping are not equal. According to Figure 15, when the damping coefficient falls within the range of 0.03 to 0.1, the jumping of the balloon predominantly exhibits a chaotic nature. However, when the damping coefficient lies between 0.1 and 0.15, the jumping of the balloon mainly shows periodic behavior.

### 4.6. Effect of Amount of Gaseous Substance

**Figure 16:**
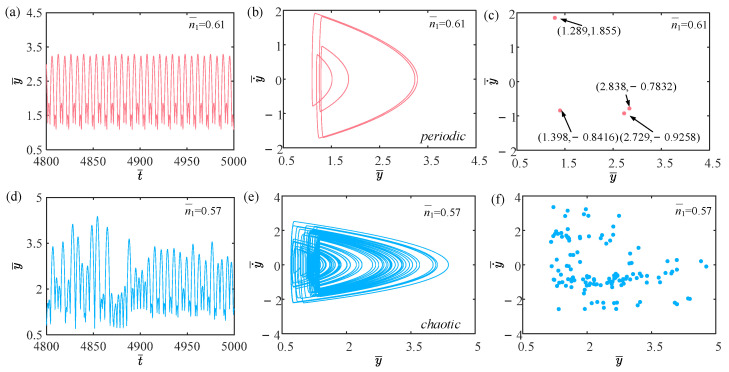
Effects of different dimensionless amounts of gaseous substance on the self-sustained jumping of the balloon. (**a**) Time history curve at ${\bar{n}}_{1}=0.61$. (**b**) Phase diagram at ${\bar{n}}_{1}=0.61$. (**c**) Poincare map at ${\bar{n}}_{1}=0.61$. (**d**) Time history curve at ${\bar{n}}_{1}=0.57$. (**e**) Phase diagram with ${\bar{n}}_{1}=0.57$. (**f**) Poincare map at ${\bar{n}}_{1}=0.57$.

**Figure 17:**
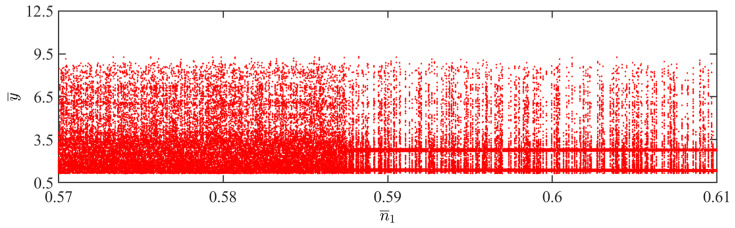
Bifurcation diagram with varying dimensionless amounts of gaseous substance.

This section explores how the amount of gaseous substance affects the self-sustained jumping of the LCE balloon, while keeping the other system parameters set as follows: $C_{0}=0.4$, $\bar{I}=0.5$, ${\bar{V}}_{L}=0.8$, ${\bar{P}}_{ext}=0.3$, $\bar{\beta }=0.05$, ${\bar{\rho }}_{L}=1$, $\bar{\alpha }=0.1$, $\bar{E}=10,000$, $\bar{g}=0.8$, and ${\bar{R}}_{p}=1.2$. Figure 16 presents the time history curves, phase diagrams, and Poincare maps for two distinct dimensionless amounts of gaseous substance, accompanied by the bifurcation diagram for the varying amounts of gaseous substance between 0.57 and 0.61 given in Figure 17. Video S8 demonstrates the effect of the amount of gaseous substance on the jumping mode of the LCE balloon. The analysis of the calculated results is similar to Section 4.1. In Figure 16a–c, when the amount of gaseous substance is 0.61, the balloon jumps alternately between four amplitudes, the trajectories on the phase diagram are regular, and there are only four fixed points on the Poincare map. These all demonstrate the periodic jumping of the balloon. Whereas for Figure 16d–f, when the amount of gaseous substance is 0.57, the jumping of the balloon has no stable amplitude, there are no repeated trajectories on the corresponding phase diagram, and the points on the corresponding Poincare diagram are random, all suggesting the chaotic jumping of the balloon.

The above transition in the jumping modes is attributed to variations in the amount of gaseous substance, which cause variations in the balloon radius and subsequently alter the Hertz contact force. The energy imbalance between the energy compensation generated by the Hertz contact force and the energy dissipated by the system damping is responsible for the emergence of chaos. It is evident from the bifurcation diagram in Figure 17 that as the amount of gaseous substance varies, the jumping mode of the balloon experiences frequent transitions between periodicity and chaos.

### 4.7. Effect of LCE Mass Density

**Figure 18:**
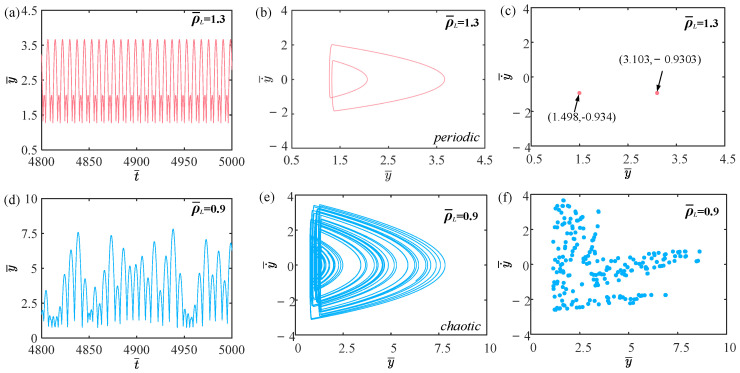
Effects of different dimensionless LCE mass densities on the self-sustained jumping of the balloon. (**a**) Time history curve at ${\bar{\rho }}_{L}=1.3$. (**b**) Phase diagram at ${\bar{\rho }}_{L}=1.3$. (**c**) Poincare map at ${\bar{\rho }}_{L}=1.3$. (**d**) Time history curve at ${\bar{\rho }}_{L}=0.9$. (**e**) Phase diagram with ${\bar{\rho }}_{L}=0.9$. (**f**) Poincare map at ${\bar{\rho }}_{L}=0.9$.

**Figure 19:**
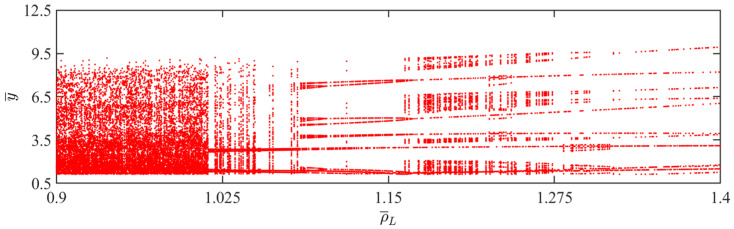
Bifurcation diagram with varying dimensionless LCE mass densities.

In this section, the primary focus is on the influence of LCE mass density on the self-sustained jumping of the LCE balloon, while the remaining system parameters are set as follows: $C_{0}=0.4$, $\bar{I}=0.5$, ${\bar{V}}_{L}=0.8$, ${\bar{P}}_{ext}=0.3$, $\bar{\beta }=0.05$, ${\bar{n}}_{1}=0.6$, $\bar{\alpha }=0.1$, $\bar{E}=10,000$, $\bar{g}=0.8$, and ${\bar{R}}_{p}=1.2$. In Figure 18, the time history curves, phase diagrams, and Poincare maps for two distinct dimensionless LCE mass densities can be found, along with the corresponding bifurcation diagram as the LCE mass density ranges from 0.9 to 1.3 being provided in Figure 19. Video S9 demonstrates the effect of LCE mass density on the jumping mode of the LCE balloon. The analysis is still similar to Section 4.1. It is evident from Figure 18a–c that when the LCE mass density is 1.3, the balloon sustains a stable amplitude, the phase diagram displays two consistent trajectories, and the Poincare maps exhibits two fixed points. Summarily, the balloon sustains a stable periodic jumping. As described in Figure 18d–f, when the LCE mass density is 1.3, there is a lack of a stable amplitude on the time history curve, no apparent periodicity in the trajectories on the phase diagram, and a random scattering of points on the Poincare map. These are indicative of a chaotic jumping of the balloon.

The transition of the jumping mode can be understood as the variation rate of the balloon radius altering with the LCE mass density, thereby affecting the magnitude of the Hertz contact force. Chaos arises when the negative work dissipated by the system damping is not balanced by the net work done by the Hertz contact force. In accordance with Figure 19, the jumping mode of the balloon is periodic when the LCE mass density is between 1.07–1.1 and 1.2–1.3. In other intervals, the jumping mode of the balloon switches frequently.

### 4.8. Effect of Beating Damping Coefficient

**Figure 20:**
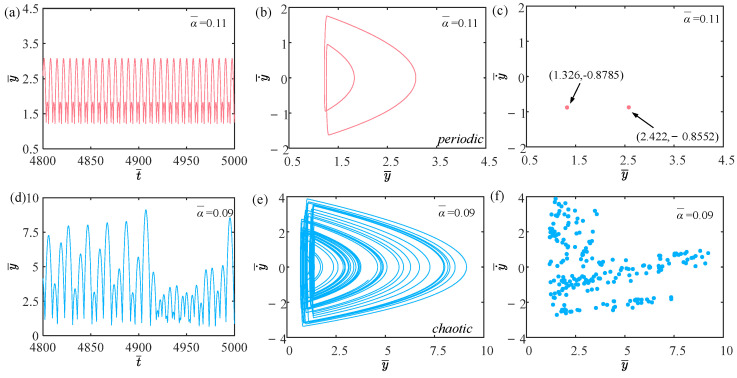
Effects of different dimensionless beating damping coefficients on the self-sustained jumping of the balloon. (**a**) Time history curve at $\bar{\alpha }=0.11$. (**b**) Phase diagram at $\bar{\alpha }=0.11$. (**c**) Poincare map at $\bar{\alpha }=0.11$. (**d**) Time history curve at $\bar{\alpha }=0.09$. (**e**) Phase diagram with $\bar{\alpha }=0.09$. (**f**) Poincare map at $\bar{\alpha }=0.09$.

**Figure 21:**
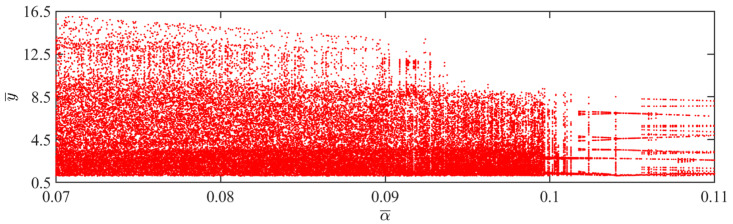
Bifurcation diagram with varying dimensionless beating damping coefficients.

We will primarily discuss the effect of the beating damping coefficient on the self-sustained jumping of the LCE balloon in this section, with other parameters being set as $C_{0}=0.4$, $\bar{I}=0.5$, ${\bar{V}}_{L}=0.8$, ${\bar{P}}_{ext}=0.3$, $\bar{\beta }=0.05$, ${\bar{n}}_{1}=0.6$, ${\bar{\rho }}_{L}=1$, $\bar{E}=10,000$, $\bar{g}=0.8$, and ${\bar{R}}_{p}=1.2$. The time history curves, phase diagrams, and Poincare maps for two distinct dimensionless beating damping coefficients are given in Figure 20, and the bifurcation diagram as the beating damping coefficient ranges from 0.07 to 0.11 is displayed in Figure 21. Video S10 demonstrates the effect of beating damping coefficient on the jumping mode of the LCE balloon. The outcome analysis remains similar to Section 4.1. As illustrated in Figure 20a–c, when the beating damping coefficient is equal to 0.11, the balloon alternates between two amplitudes, the phase diagram exhibits a stable periodic trajectory, and there are only two fixed points on the Poincare map. These observations suggest that the jumping of the balloon is periodic. According to Figure 20d–f, when the beating damping coefficient is equal to 0.09, the balloon does not behave with stable amplitudes, and the phase diagram and Poincare map do not show predictable behavior, indicating that the jumping of the balloon is chaotic.

Obviously, variations in the beating damping coefficient will affect the magnitude of the Hertz contact force. The appearance of chaos comes when the net work done by the Hertz contact force differs from the negative work dissipated by the system damping. From Figure 21, it is evident that the jumping mode of the balloon does not exhibit any regularity with the varying beating damping coefficients.

### 4.9. Effect of Elastic Modulus

**Figure 22:**
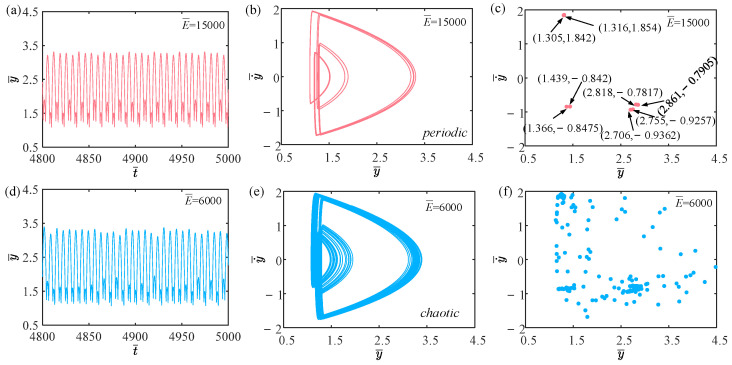
Effects of different dimensionless elastic moduli on the self-sustained jumping of the balloon. (**a**) Time history curve at $\bar{E}=15,000$. (**b**) Phase diagram at $\bar{E}=15,000$. (**c**) Poincare map at $\bar{E}=15,000$. (**d**) Time history curve at $\bar{E}=6000$. (**e**) Phase diagram with $\bar{E}=6000$. (**f**) Poincare map at $\bar{E}=6000$.

**Figure 23:**
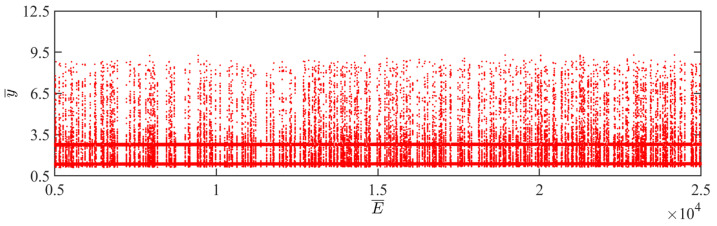
Bifurcation diagram with varying dimensionless elastic moduli.

In this section, we primarily discuss the effect of the elastic modulus on the self-sustained jumping of the LCE balloon, with the remaining system parameters set as $C_{0}=0.4$, $\bar{I}=0.5$, ${\bar{V}}_{L}=0.8$, ${\bar{P}}_{ext}=0.3$, $\bar{\beta }=0.05$, ${\bar{n}}_{1}=0.6$, ${\bar{\rho }}_{L}=1$, $\bar{\alpha }=0.1$, $\bar{g}=0.8$, and ${\bar{R}}_{p}=1.2$. Figure 22 illustrates the time history curves, phase diagrams, and Poincare maps for the two distinct dimensionless elastic moduli, and Figure 23 depicts the bifurcation diagram of the elastic modulus varying from 5000 to 25,000. The analysis is still similar to Section 4.1. Video S11 demonstrates the effect of elastic modulus on the jumping mode of the LCE balloon. As displayed in Figure 22a–c, the jumping of the balloon exhibits stable amplitudes, and the phase diagram and Poincare map show regular patterns, indicating that the jumping of the balloon is periodic. From Figure 22d–f, it can be observed that there are no stable amplitudes in the time history curve, and the phase diagram and Poincare map show irregular patterns. These observations reveal that the jumping of the balloon is chaotic.

Since the elastic modulus affects the stiffness of the elastic substrate and the LCE balloon, it ultimately influences the magnitude of the Hertz contact force. Chaos occurs when the net work done by the Hertz contact force is not sufficient to complement the damping dissipation of the system. From Figure 23, it can be seen that there is no clear boundary between the periodic jumping and chaotic jumping of the balloon as the elastic modulus varies.

### 4.10. Effect of Gravitational Acceleration

**Figure 24:**
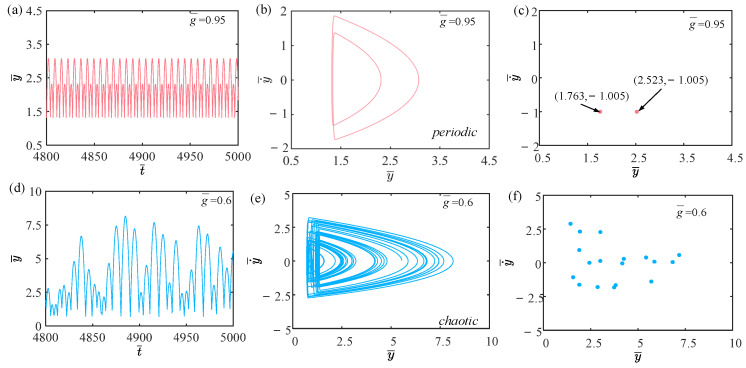
Effects of different dimensionless gravitational accelerations on the self-sustained jumping of the balloon. (**a**) Time history curve at $\bar{g}=0.95$. (**b**) Phase diagram at $\bar{g}=0.95$. (**c**) Poincare map at $\bar{g}=0.95$. (**d**) Time history curve at $\bar{g}=0.6$. (**e**) Phase diagram with $\bar{g}=0.6$. (**f**) Poincare map at $\bar{g}=0.6$.

**Figure 25:**
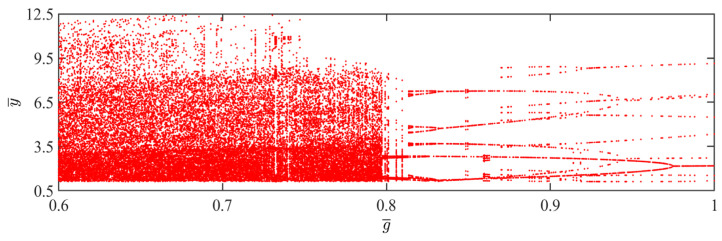
Bifurcation diagram with varying dimensionless gravitational acceleration.

In this section, we discuss the influence of gravitational acceleration on the self-sustained jumping of the LCE balloon, where the other system parameters are set as: $C_{0}=0.4$, $\bar{I}=0.5$, ${\bar{V}}_{L}=0.8$, ${\bar{P}}_{ext}=0.3$, $\bar{\beta }=0.05$, ${\bar{n}}_{1}=0.6$, ${\bar{\rho }}_{L}=1$, $\bar{\alpha }=0.1$, $\bar{E}=10,000$ and ${\bar{R}}_{p}=1.2$. Figure 24 depicts the time history curves, phase diagrams, and Poincare maps for two distinct dimensionless gravitational accelerations. Additionally, a bifurcation diagram showing the variation of gravitational acceleration within the range of 0.6 to 1 is plotted in Figure 25. Video S12 demonstrates the effect of gravitational acceleration on the jumping mode of the LCE balloon. The analysis of the calculated results is similar to Section 4.1. As observed in Figure 24a–c, the balloon jumps with a stable amplitude, the phase diagram displays two periodic trajectories, and the Poincare map has only two fixed points. These demonstrate that the balloon is undergoing a periodic jumping. In Figure 24d–f, the jumping amplitude of the balloon is not regular, and the trajectories on the phase diagram and the points’ positions on the Poincare map are unpredictable. It can be inferred that the jumping of the balloon is chaotic.

Due to the variations in gravitational acceleration affecting the upward jumping of the balloon, which in turn alter the jumping amplitude of the balloon, chaos occurs when the negative work done by air resistance during the motion is not equal to the net work compensated by the system. According to Figure 25, when the gravitational acceleration varies between 0.81 and 1, the jumping of the balloon is periodic. When the gravitational acceleration varies between 0.6 and 0.81, the jumping of the balloon is primarily chaotic.

### 4.11. Effect of Painting Radius

**Figure 26:**
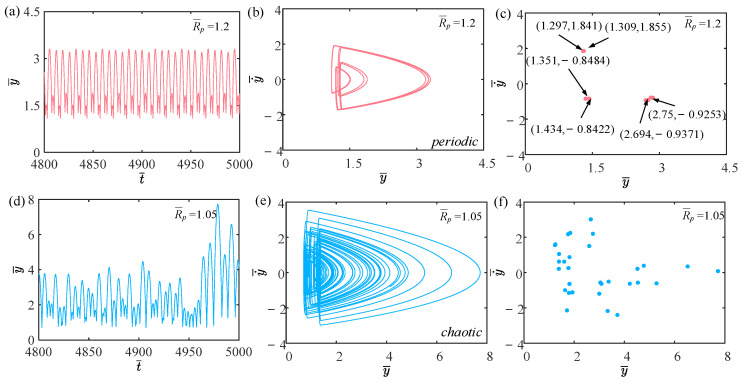
Effects of different dimensionless painting radii on the self-sustained jumping of the balloon. (**a**) Time history curve at ${\bar{R}}_{p}=1.2$. (**b**) Phase diagram at ${\bar{R}}_{p}=1.2$. (**c**) Poincare map at ${\bar{R}}_{p}=1.2$. (**d**) Time history curve at ${\bar{R}}_{p}=1.05$. (**e**) Phase diagram with ${\bar{R}}_{p}=1.05$. (**f**) Poincare map at ${\bar{R}}_{p}=1.05$.

**Figure 27:**
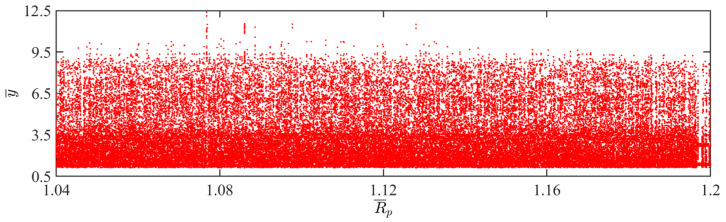
Bifurcation diagram with varying dimensionless painting radius.

In this section, we will discuss the effect of the painting radius on the self-sustained jumping of the LCE balloon. Except for the painting radius, the settings for the other parameters are as follows: $C_{0}=0.4$, $\bar{I}=0.5$, ${\bar{V}}_{L}=0.8$, ${\bar{P}}_{ext}=0.3$, $\bar{\beta }=0.05$, ${\bar{n}}_{1}=0.6$, ${\bar{\rho }}_{L}=1$, $\bar{\alpha }=0.1$, $\bar{E}=10,000$, and $\bar{g}=0.8$. Figure 26 plots the time history curves, phase diagrams, and Poincare maps for two distinct dimensionless painting radii. Additionally, the bifurcation diagram corresponding to the painting radius varying from 0.6 to 1 is provided in Figure 27. Video S13 demonstrates the effect of the painting radius on the jumping mode of the LCE balloon. The analysis of the calculation outcomes is similar to Section 4.1. For Figure 26a–c, when the painting radius is 1.2, the balloon jumps regularly. The trajectories on the phase diagram are regular, and there are only several fixed points on the Poincare map. These suggest the periodic jumping of the balloon. As observed in Figure 26d–f, when the painting radius is 1.05, the jumping of the balloon has no stable amplitude, there are no repeated trajectories on the phase diagram, and the points on the Poincare diagram are random, demonstrating the chaotic jumping of the balloon.

The above switching of jumping modes is owing to the change in the balloon deformation period caused by the variation in the painting radius, thus affecting the Hertz contact force. The imbalance between the work done by the Hertz contact force and the damping dissipation is responsible for the occurrence of chaos. As shown in Figure 27, as the painting radius varies, no clear boundary is present between the periodic and chaotic jumping of the balloon.

## 5. Conclusions

A self-sustained jumping system can maintain periodic jumping and chaotic jumping under constant stimulus, which holds significant promise in actuators, medical devices, biomimetic robots, and other fields. In this paper, a light-powered self-sustained chaotic jumping LCE balloon with a self-shading effect is innovatively developed. Owing to the self-shading effect of the coating, the LCE balloon contracts and expands under steady illumination, thus maintaining a self-sustained jumping. The numerical results demonstrate that two typical jumping modes of the LCE balloon are present, namely, periodic jumping and chaotic jumping. The corresponding mechanisms underlying the self-sustained periodic jumping and chaotic jumping are explained according to the relationship between the work done by the contraction and expansion of the LCE balloon and the energy dissipated by the system damping. The self-sustained jumping of the LCE balloon can be triggered by controlling key system parameters, such as the light intensity, contraction coefficient, balloon volume, external pressure, damping coefficient, etc. By adjusting several system parameters, we can control the balloon to switch jumping modes. The system can achieve self-balancing under steady and uniform illumination with full-field stability. Researchers can conduct relevant experimental studies on the basis of the theoretical research in this paper and compare with the predictions in this paper. We hope that this research provides potential applications and aid in the development of novel intelligent material-based robots, the design of new energy conversion equipment, and so on.

## Data Availability

The data that support the findings of this study are available upon reasonable request from the authors.

## Supplementary Materials

The following supporting information can be downloaded at https://www.mdpi.com/article/10.3390/polym15244651/s1, Video S1: Periodic motion mode of the LCE balloon; Video S2: Chaotic motion mode of the LCE balloon; Video S3: Effect of light intensity on the jumping mode of the LCE balloon; Video S4: Effect of contraction coefficient on the jumping mode of the LCE balloon; Video S5: Effect of balloon volume on the jumping mode of the LCE balloon; Video S6: Effect of external pressure on the jumping mode of the LCE balloon; Video S7: Effect of damping coefficient on the jumping mode of the LCE balloon; Video S8: Effect of amount of gaseous substance on the jumping mode of the LCE balloon; Video S9: Effect of LCE mass density on the jumping mode of the LCE balloon; Video S10: Effect of beating damping coefficient on the jumping mode of the LCE balloon; Video S11: Effect of elastic modulus on the jumping mode of the LCE balloon; Video S12: Effect of gravitational acceleration on the jumping mode of the LCE balloon; Video S13: Effect of painting radius on the jumping mode of the LCE balloon.
