# Supplementary material for: Self-Sustained Chaotic Jumping of Liquid Crystal Elastomer Balloon under Steady Illumination
Source: Polymers (Basel). 2023 Dec 8;15(24):4651. doi: 10.3390/polym15244651 (PMC10747744; doi:10.3390/polym15244651)
Supplement: Supplementary file 1 [file polymers-15-04651-s001.zip › polymers-2708614-supplementary/polymers-2708614-supplementary.pdf]

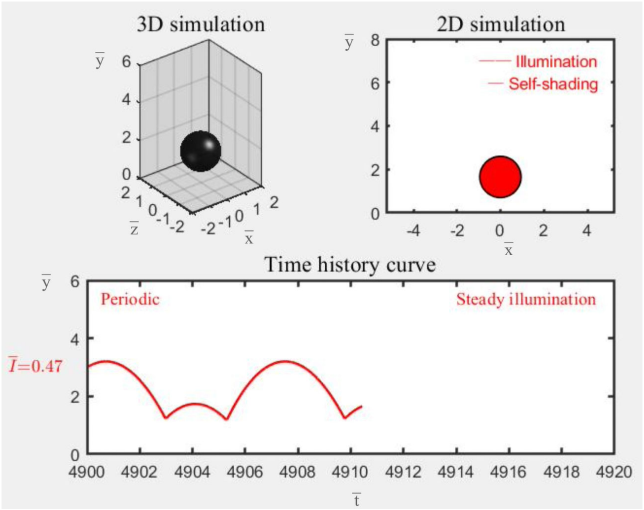

Video S**1** Periodic motion mode of the LCE balloon.

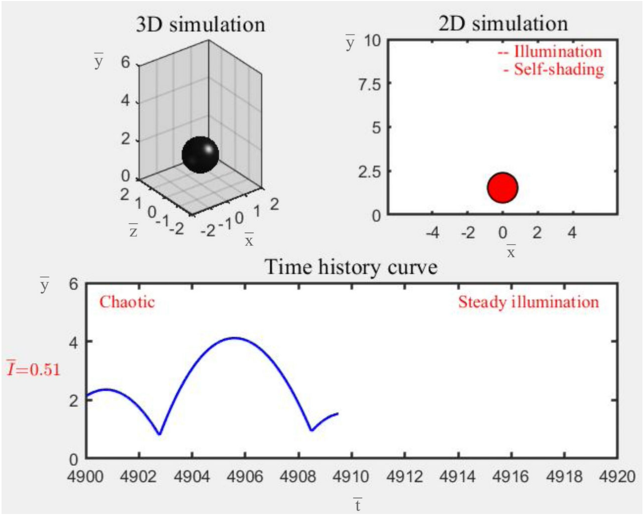

Video S2 Chaotic motion mode of the LCE balloon.

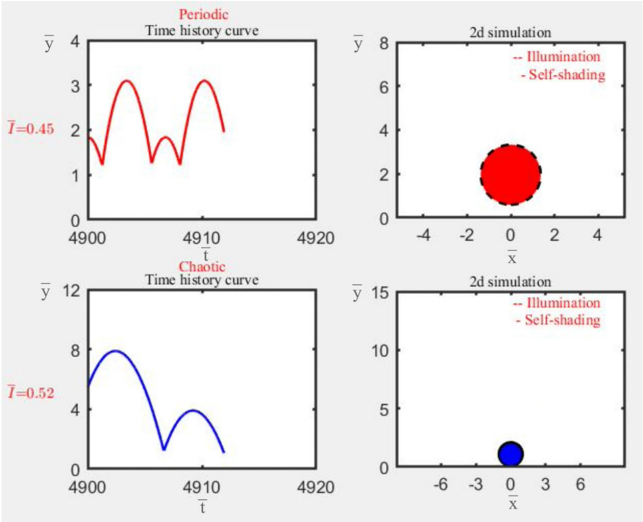

Video S3 Effect of light intensity on the jumping mode of the LCE balloon.

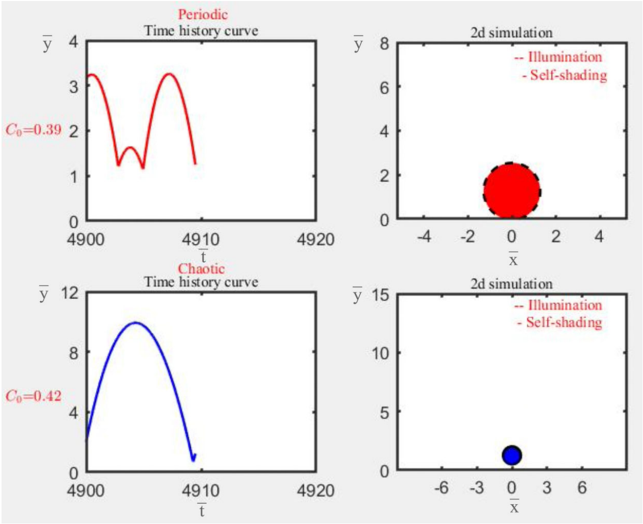

Video S4 Effect of contraction coefficient on the jumping mode of the LCE balloon.

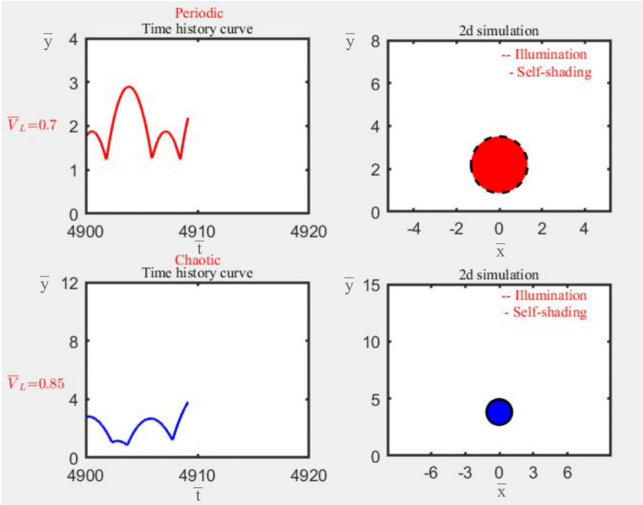

Video S5 Effect of balloon volume on the jumping mode of the LCE balloon.

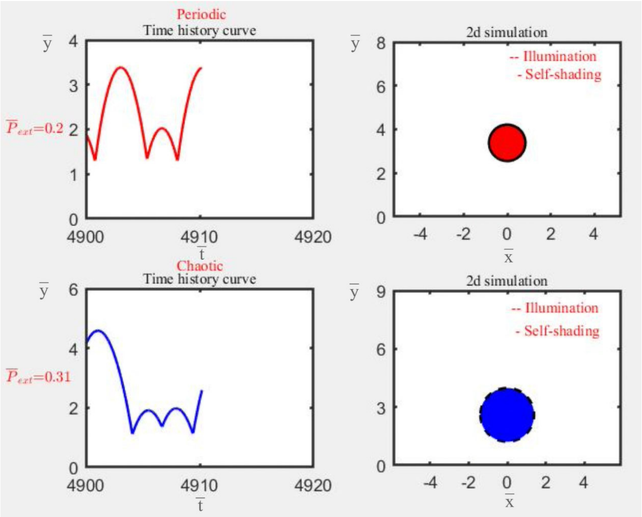

Video S6 Effect of external pressure on the jumping mode of the LCE balloon

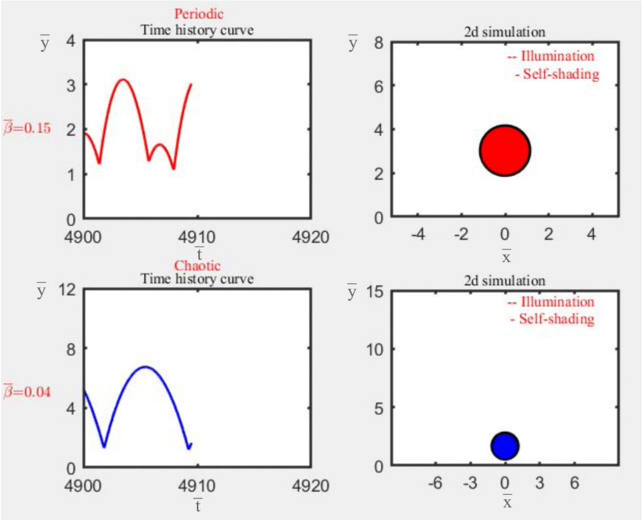

Video S7 Effect of damping coefficient on the jumping mode of the LCE balloon.

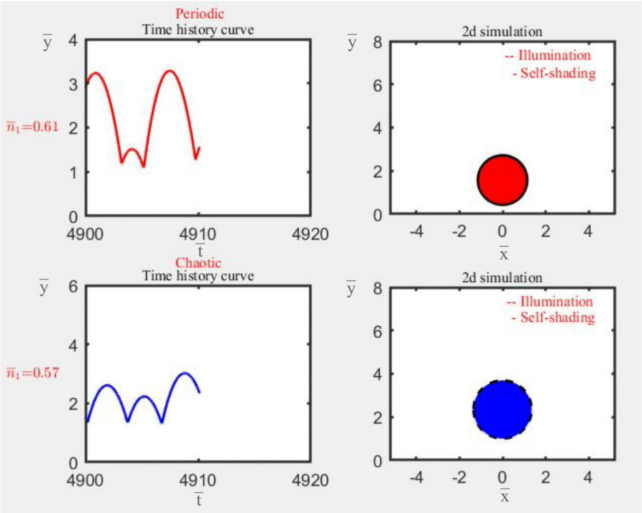

Video S8 Effect of amount of gaseous substance on the jumping mode of the LCE balloon.

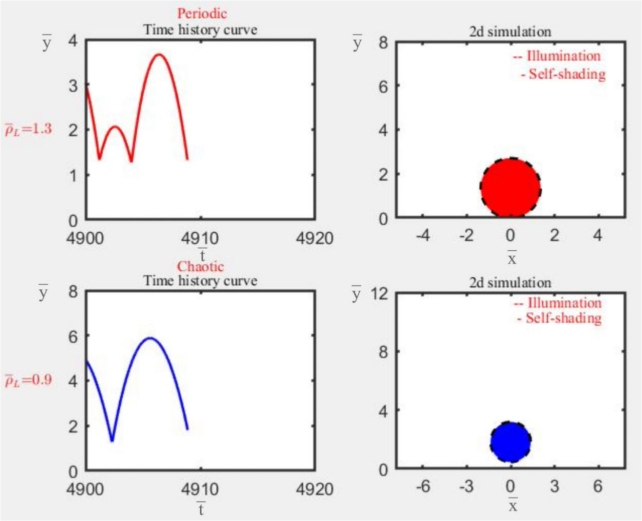

Video S9 Effect of LCE mass density on the jumping mode of the LCE balloon.

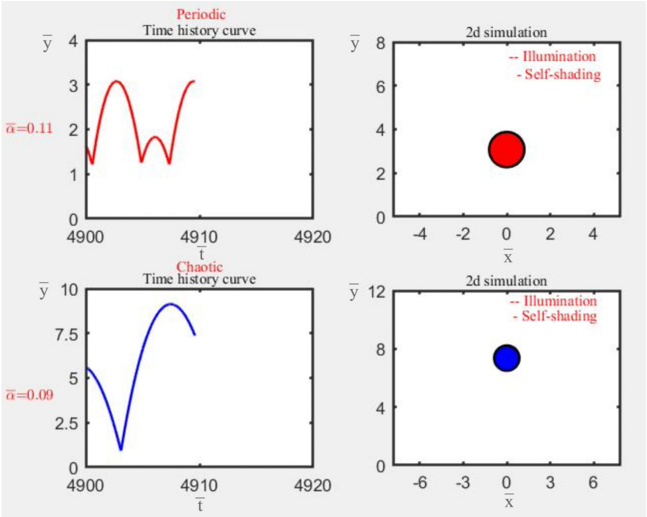

Video S10 Effect of beating damping coefficient on the jumping mode of the LCE balloon.

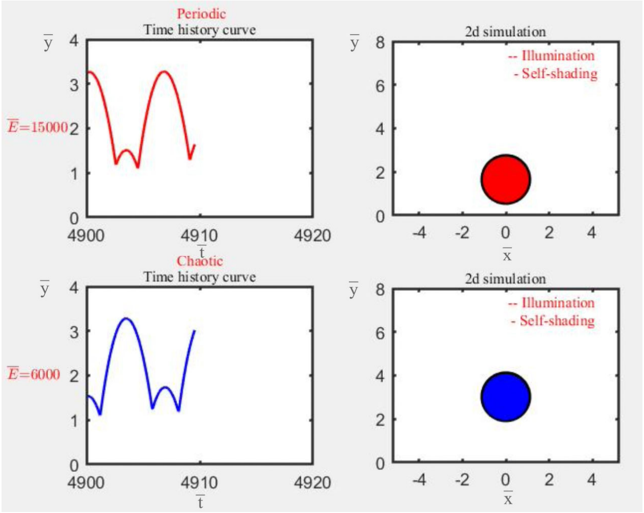

Video S11 Effect of elastic modulus on the jumping mode of the LCE balloon.

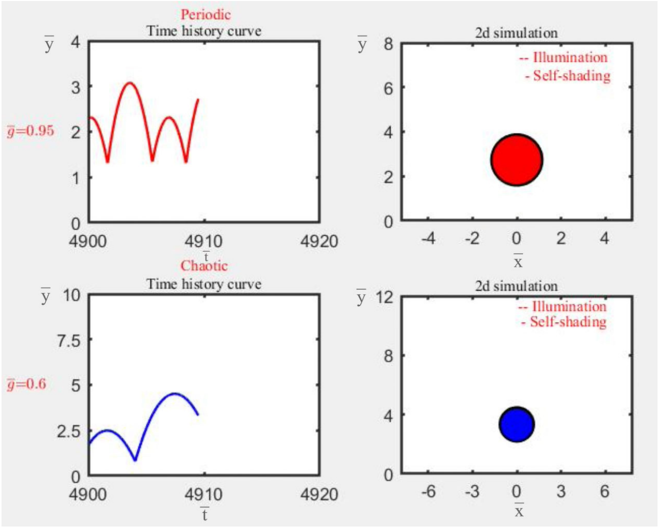

Video S12 Effect of gravitational acceleration on the jumping mode of the LCE balloon.

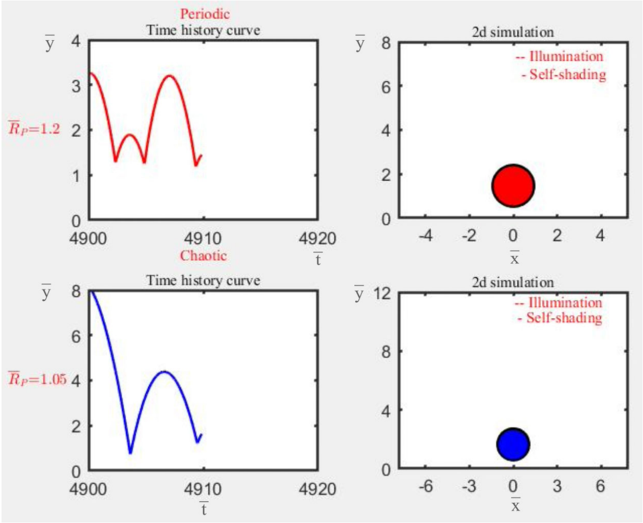

Video S13 Effect of painting radius on the jumping mode of the LCE balloon.
